# Brief Report: A Novel Sodium/Iodide Symporter Mutation, S356F, Causing Congenital Hypothyroidism

**DOI:** 10.1089/thy.2021.0478

**Published:** 2022-02-10

**Authors:** Harsh Durgia, Adeline K. Nicholas, Erik Schoenmakers, Jennifer A. Dickens, Dhanapathi Halanaik, Jayaprakash Sahoo, Sadishkumar Kamalanathan, Nadia Schoenmakers

**Affiliations:** ^1^ Department of Endocrinology, Jawaharlal Institute of Postgraduate Medical Education and Research (JIPMER), Puducherry, India.; ^2^ University of Cambridge Metabolic Research Laboratories, Wellcome Trust-Medical Research Council Institute of Metabolic Science, Addenbrooke's Hospital, Cambridge, United Kingdom.; ^3^ Cambridge Institute for Medical Research, Cambridge, United Kingdom.; ^4^ Department of Nuclear Medicine, Jawaharlal Institute of Postgraduate Medical Education and Research (JIPMER), Puducherry, India.

**Keywords:** congenital hypothyroidism, dyshormonogenesis, iodide transport, SLC5A5

## Abstract

The sodium-iodide symporter (NIS, SLC5A5) is expressed at the basolateral membrane of the thyroid follicular cell, and facilitates the thyroidal iodide uptake required for thyroid hormone biosynthesis. Biallelic loss-of-function mutations in NIS are a rare cause of dyshormonogenic congenital hypothyroidism. Affected individuals typically exhibit a normally sited, often goitrous thyroid gland, with absent uptake of radioiodine in the thyroid and other NIS-expressing tissues. We report a novel homozygous NIS mutation (c.1067 C>T, p.S356F) in four siblings from a consanguineous Indian kindred, presenting with significant hypothyroidism. Functional characterization of the mutant protein demonstrated impaired plasma membrane localization and cellular iodide transport.

## Introduction

The sodium-iodide symporter (NIS, SLC5A5) is a membrane glycoprotein expressed basolaterally in thyroid follicular epithelial cells, which mediates the active uptake and accumulation of iodide. Iodide is subsequently oxidized, organified, and incorporated into thyroid hormones. Homozygous NIS mutations are an uncommon cause of dyshormonogenic congenital hypothyroidism (CH) for which the clinical presentation and the underlying molecular basis are variable. To date, 13 functionally characterized missense mutations have been identified in ∼30 kindreds worldwide (1,2). In this study, we report and characterize a novel homozygous NIS missense mutation underlying CH in four siblings of Indian origin.

## Methods

Methods are detailed in Supplementary Data. Studies were undertaken with written informed consent from the patients or their parents, either under clinical auspices or as part of an ethically-approved protocol (Cambridge South, MREC 98/5/24).

## Case Reports

The female proband was born to consanguineous Indian parents and, through lack of a local newborn CH screening program, presented with clinically evident hypothyroidism aged 5 months. Variable compliance with levothyroxine resulted in re-presentation aged 16 years with growth retardation (height below the third percentile) dry skin, and delayed relaxation of the ankle reflexes. Biochemistry confirmed suboptimally treated primary hypothyroidism: thyrotropin (TSH) 137 mU/L (normal range [NR] 0.35–5.5), free thyroxine 0.47 mg/dL (NR 0.89–1.76).

**FIG. 1.**
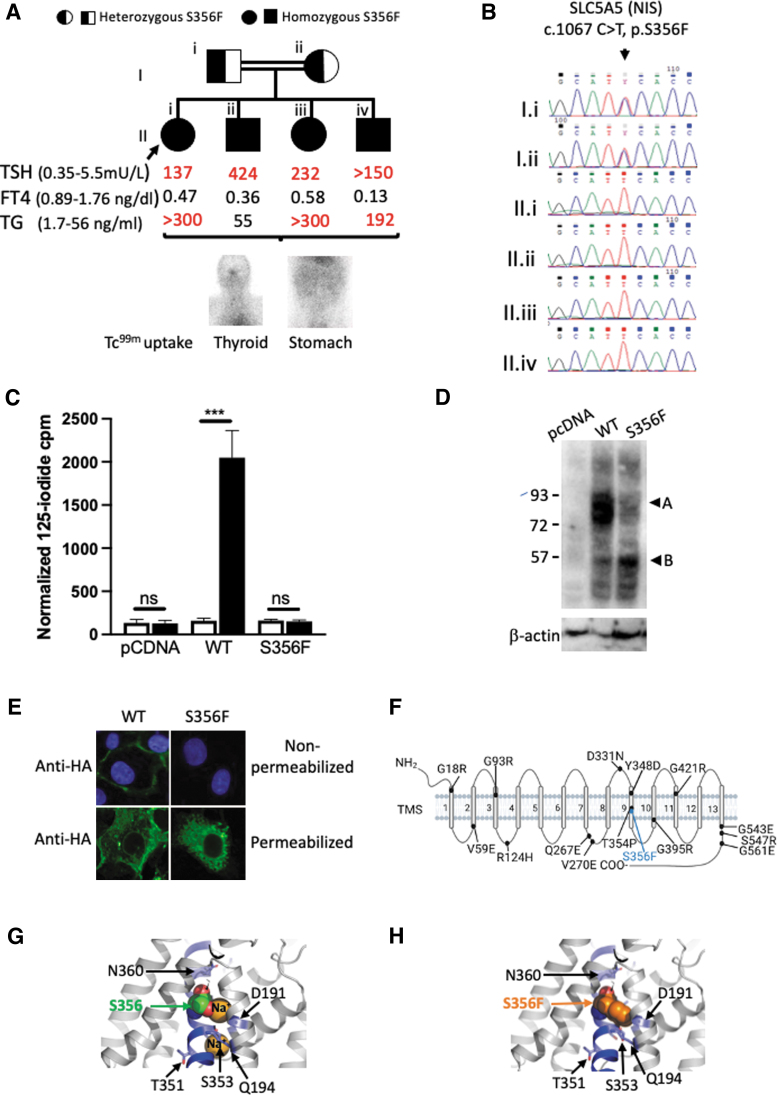
(**A**) Pedigree showing homozygosity (black) and heterozygosity (black and white) for the NIS p.S356F mutation, in a consanguineous family from India. Corresponding thyroid hormones (measured on ADVIA Centaur XP Immunoassay System) and TG (measured on IMMULITE 1000 Immunoassay System) measured while on suboptimal levothyroxine replacement (100 mcg daily), aged 16, 14, 12, and 8 years, respectively, are aligned to the respective individual. Representative Tc-99m pertechnetate scan images for thyroid and stomach are shown hereunder; there is no uptake of tracer into either organ. (**B**) Representative sequencing chromatogram for each of the four siblings (all homozygous for p.S356F NIS) and their heterozygous parents, showing the relevant homo- or heterozygous nucleotide substitution (c.1067C>T). (**C**) Graph showing mean normalized ^125^I uptake in COS-7 cells transfected with pcDNA, WT, or p.S356F NIS, in the presence (white) or absence (black) of 100 μM perchlorate. Data are expressed in counts per minute standardized to transfection efficiency as assessed by B-galactosidase assay and represent the mean and SEM values obtained of three independent experiments performed in triplicate. *p*-Values were calculated using ANOVA and Tukey's *post hoc* test ***p < 0.0005. (**D**) Representative Western blot analysis of whole cell lysates of COS-7 cells transiently transfected with HA-WT NIS (WT), HA-S356F NIS (S356F), or empty vector (pcDNA) and probed with anti-HA and anti-beta actin antibodies. (**A**) Fully glycosylated mature NIS monomer (∼75–90 kDa), (**B**) unglycosylated immature NIS monomer (∼55 kDa). (**E**) Representative immunofluorescence analysis of COS-7 cells transiently transfected with HA-WT NIS (WT), HA-S356F NIS (S356F), and probed with anti-HA antibodies in the nonpermeabilized and permeabilized state (green). The nuclei of nonpermeabilized cells are stained with DAPI (blue). (**F**) Schematic of the secondary structure of NIS with TMS numbered adjacent to each domain. The structure is annotated with previously reported NIS missense mutations and the position of S356 is depicted in blue. This figure was created in Biorender.com. **(G, H)** SLC5A5 3D structure homology models generated using PHYRE2 Protein Fold Recognition Server showing a detailed view of WT NIS (**G**) with the position of Ser356 (green) in transmembrane helix 9 (blue) orientation toward Na+ atoms (orange) and other amino acids reported to be involved in Na^+^ binding (3). (**H**) Model of Ser356Phe NIS: the mutation is predicted to impede Na^+^ binding due to steric hindrance. Moreover, Ser356 is polar, and the apolar and larger Phe will disrupt the local structure, potentially destabilizing the overall structure of NIS. ANOVA, analysis of variance; COS-7, CV-1 (simian) in Origin, carrying SV40 genetic material; DAPI, 4′,6-diamidino-2-phenylindole; HA, human influenza hemagglutinin; NIS, sodium-iodide symporter; SEM, Standard error of the mean; TG, thyroglobulin; TMS, transmembrane segments; WT, wild-type. Color images are available online.

An elevated thyroglobulin level (>300 ng/mL, NR 1.7–56) confirmed the presence of a thyroid gland; however, undetectable uptake of technetium-99m pertechnetate (a NIS substrate) in thyroid, salivary glands, and stomach supported an iodide transport defect (Fig. 1A). Her three younger siblings were diagnosed with hypothyroidism between the ages of 4 and 7 months with similar biochemistry and scintigraphy results; there was no clinical evidence of goiter in any of the children and both parents were biochemically euthyroid (Fig. 1A).

## Results

Sanger sequencing of the coding exons of *SLC5A5* (RefSeq: NM_000453.3) revealed a homozygous missense mutation (c.1067C>T, p.S356F), segregating with hypothyroidism in the family (Fig. 1B), which was absent from the gnoMAD database and predicted to be pathogenic by PolyPhen-2: 0.999 (scale: 0: benign, 1.0 probably damaging), MutationTaster2, *p* = 0.99 (*p*-values close to 1 indicate a high confidence prediction) and sorting intolerant from tolerant: 0.00 (scale 0: deleterious, 1: tolerated).

COS-7 cells were transiently transfected to compare the expression and activity of the wild-type (WT) and S356F mutant NIS protein. Human influenza hemagglutinin (HA) tagged-S356F NIS demonstrates negligible perchlorate-sensitive iodide accumulation compared with cells expressing HA-WT NIS (Fig. 1C). Western blotting shows decreased levels of fully glycosylated HA-S356F NIS polypeptide compared with HA-WT NIS (∼75–90 kDa, Fig. 1D) and only HA-WT NIS is clearly detectable at the plasma membrane (PM) (Fig. 1E), although both HA-WT and HA-S356F NIS are present in permeabilized cells (Fig. 1E).

## Discussion

NIS loss-of-function is associated with a heterogenous clinical phenotype with variable goitrogenesis and onset of hypothyroidism. Deleteriousness of the mutation, and residual NIS function *in vivo* correlate to some extent with age at which hypothyroidism manifests (1). Our patients had undetectable Tc-99m pertechnetate uptake, suggesting severe NIS functional impairment, consistent with their significant, early-onset hypothyroidism. Surprisingly, despite marked, and presumably prolonged TSH elevation, this was nongoitrous. However, goiter correlates poorly with NIS genotype and our patients' iodide status was not assessed, which may also have mitigated goitrogenesis, currently being replete in most of India.

The structure of NIS comprises an extracellular amino terminus, 13 transmembrane segments (TMS), and a cytosolic carboxy terminus (Fig. 1F), and previously characterized CH-associated NIS mutations affect PM expression and/or transporter function. The mutation described here, p.S356F, is the third reported CH-associated mutation in TMS 9, after p.T354P and p.Y348D. Both p.T354P and p.S356F alter one of several β-OH group-containing residues, thought to be essential for Na^+^ binding and/or translocation, without directly impacting PM expression, as determined by characterization of p.T354P and additional, artificial T354, and S356 mutations (2,3).

In contrast, Y348 is not known to be directly involved in Na+ transport and PM-translocation; however, p.Y348D NIS exhibits impaired transporter activity, maturation, and trafficking, being only partially glycosylated and retained intracellularly (2). In both Y348D and S356F the replacement of a large hydrophobic residue by a smaller hydrophilic residue, or *vice versa*, probably results in impaired protein folding. This has similar deleterious effects on NIS maturation and PM targeting and (although p.S356F intrinsic activity has not been investigated) results in a clear absence of PM iodide transport explaining the lack of thyroidal iodide transport in the patient (2, Fig. 1A, C–E, G, H).

NIS mutations are uncommon and, to the best of our knowledge, this is the first report of a deleterious NIS missense mutation associated with hypothyroidism in individuals of Indian race/ethnicity. The prevalence of permanent CH in the Indian subcontinent is high (1:1130), of which a significant proportion is due to dyshormonogenesis (46%); however, its genetic determinants are largely uncharacterized (4). Our findings suggest that NIS mutations contribute to CH in India and confirm the importance of residue S356 for normal NIS function *in vivo.*

## Supplementary Material

Supplemental data
