# Supplementary material for: Brief Report: A Novel Sodium/Iodide Symporter Mutation, S356F, Causing Congenital Hypothyroidism
Source: Thyroid. 2022 Feb 10;32(2):215–8. doi: 10.1089/thy.2021.0478 (PMC8861941; doi:10.1089/thy.2021.0478)
Supplement: Supplemental data [file Suppl_Data.docx]

**Brief Report: A Novel Sodium/Iodide Symporter Mutation S356F Causing Congenital Hypothyroidism. (H. Durgia et al).**

**Supplementary Data**

**Methods**

**Ethics statement**

Studies were undertaken with written informed consent from the patients or their parents, either under clinical auspices or as part of an ethically-approved protocol (Cambridge South, MREC 98/5/24). The family consented to publication of their case reports in a scientific journal.

**Thyroid imaging**

Thyroid pertechnetate scans were performed at diagnosis using a Symbia T6 dual-head gamma camera after an intravenous injection of 99m-technetium pertechnetate (5mCi), obtaining standard anterior and lateral images and recording the size and location of areas of 99mTcO_4_ uptake.

**Statistical analysis**

Statistical analyses were performed using Prism, version 8.4.3, June 10 2020 (GraphPad Software, San Diego, California USA, www.graphpad.com).

**Biochemical assays**

Serum TSH and T4/FT4 were measured locally using the Advia Centaur XP chemiluminescent immunoassay system while thyroglobulin was measured with Immulite 1000 chemiluminescent immunoassay system. All values were compared with kit reference ranges.

**Sanger sequencing**

Genomic DNA was extracted from peripheral blood leukocytes using standard techniques. All coding exons and exon/intron boundaries for SLC5A5 were amplified by PCR using specific primers (available on request). Family members were genotyped for the specific mutation identified in the Proband. PCR products were sequenced using the BigDye Terminator v3.1 Cycle Sequencing Kit (Applied Biosystems, Foster City, USA) and 3730 DNA Analyzer (Applied Biosystems). Variants were described using the systematic nomenclature approved by the Human Genome Variation Society (HGVS; www.hgvs.org/mutnomen). Nucleotide numbering starts from the A (+1) of the translation initiation codon (ATG) of the NCBI reference sequences below: ENST00000222248.4, NM_000453.3, NP_000444.1 (SLC5A5)

**Variant analysis**

Variants with global minor allele frequency (MAF) <0.01 and maximal minor allele frequency <0.02 in the gnoMAD database were assessed using the in silico pathogenicity prediction tools PolyPhen-2, SIFT and MutationTaster2. (1-3). Numerical classification by these tools indicates the following: PolyPhen-2: 0.0: benign; 1.0: damaging; MutationTaster2: p-values close to 1 indicate a high confidence level in the prediction, SIFT: 0.0: damaging; 1.0: tolerated (1-3).

**Cloning**

Human SLC5A5 with an extracellular, N-terminal HA-tag in a pcDNA3 vector was a kind gift from Dr N Carrasco, Department of Cellular and Molecular Physiology, Yale School of Medicine, New Haven, Connecticut. The c.1067C>T mutation was generated by site directed mutagenesis of the wild type (WT) human SLC5A5 cDNA.

**Iodide uptake**

COS-7 cells grown in 24 well plates in DMEM (+ 10% FBS, 1% PSF and 1% L-glutamine) were co-transfected with 250ng of either HA-epitope-tagged WT SLC5A5, HA-epitope-tagged S356F SLC5A5 or empty vector (pcDNA3) and 50ng internal control plasmid BOS-beta-galactosidase using lipofectamine 2000 (ThermoFisher), as per the manufacturers protocol. 24 hours after transfection, cells were incubated for 1 h at 37^o^C, with 5% CO_2_, in 0.5ml HBSS (Sigma H9269) containing 0.07microcurie Na [^125^I] (PerkinElmer, NEZ033H001MC ) and 1 μM NaI, in the presence or absence of the NIS inhibitor sodium perchlorate (100μM). Cells were then washed in HBSS and lysed (25mM glycyl-glycine, 15mM MgSO_4_, 4mM EGTA, 1% Triton, 1mM DTT) and radioactivity measured using the Topcount microplate scintillation counter (Perkin Elmer). Radioiodine uptake was expressed as counts/min adjusted for transfection efficiency as assessed by β-galactosidase activity.

**Western Blot**

COS-7 cells, cultured in a 6 well plate in DMEM + 10% FBS, 1% PSF and 1% L-glutamine, were transfected with 500ng HA-epitope-tagged WT or p.S356F SLC5A5 or empty vector (pcDNA3) using lipofectamine 2000 (ThermoFisher). 24 hours after transfection, cells were lysed (50mM Tris HCl pH 7, 150mM NaCl, 0.5% SDS, 1% NP40, 1mM DTT complete protease inhibitor cocktail, 0.25mU benzonase (Novagen)). Following denaturation at 65^o^C for 5mins, 50μg lysate was run on an 8% Bis-Tris gel (Invitrogen) in MOPS buffer. Expression of HA-NIS was analysed by Western blotting using an anti-HA antibody (1 in 1000, HA.11, Covance) and expression of beta-actin was analyzed using an anti-human beta-actin antibody, (1 in 2000, Abcam, ab8227).

**Membrane localization**

COS-7 cells, cultured in a 96 well plate in DMEM + 10% FBS, 1% PSF and 1% L-glutamine, were transfected with 50ng HA-epitope-tagged WT or p.S356F SLC5A5 or empty vector (pcDNA3) using lipofectamine 2000 (ThermoFisher). 24 hours after transfection, cells were fixed in 10% NBF. Cells (either non-permeabilized or permeabilized using PBS/0.5% Triton-X100) were incubated with anti-HA antibody (1 in 1000, HA.11, Covance) then washed with PBS/0.2% BSA and incubated with Alexa Fluor 488-conjugated anti-mouse IgG (A21202, Invitrogen). Images were captured using the Opera Phenix High Content Screening System, using a 40x IMM NA1.1 lens, exciting with 405nm and 488nm lasers for DAPI and AF488, respectively. The camera was set at 100ms exposure foreach channel. 10 regions were imaged per well. The focal plane was maintained by a hardware focus drive. The Opera Phenix Images in 96 well format, and acquires unbiased images regions across the plate, maintaining the same image settings throughout. Representative images were selected for Figure 1 E.

**Homology Modelling**

SLC5A5 3D structure homology models were generated using PHYRE2 Protein Fold Recognition Server (4). The figures were generated with MacPyMOL Molecular Graphics System, Schrödinger, LLC. In the current working models proposed (5-8) amino acid S356 (green) is situated in transmembrane helix 9 (blue) in a region involved in either Na+/I- binding and/or translocation. In the model shown here, 2 Na atoms (orange) are positioned similar to their position in the Na+-coupled sialic acid symporter (PDB 5NVA) by superposition of the model and the 5NVA structure.

**Secondary Structure of NIS**

The schematic illustrating the secondary structure of NIS with reported missense mutations is based on the predicted secondary structure previously reported by Levy et al (9). Previously reported mutations are described in (10, review) and in the following papers (11-16).

**References**

1. Schwarz, J. M., Cooper, D. N., Schuelke, M., et al. 2014 .MutationTaster2: mutation prediction for the deep-sequencing age. Nat Methods.11:361-362.

2. Adzhubei IA, Schmidt S, Peshkin L, et al. 2010 A method and server for predicting damaging missense mutations. Nat Methods.74:248–249.

3. Kumar, P., Henikoff, S., Ng, P. C. 2009.Predicting the effects of coding non-synonymous variants on protein function using the SIFT algorithm. Nat Protoc. 4:1073–1081.

4. Kelley LA, Mezulis S, Yates CM, et al 2015 The Phyre2 web portal for protein modeling, prediction and analysis Nature Protocols 10; 845-858

5. Wahlgren, WY, Dunevall E, North RA, et al 2018 Substrate-bound outward-open structure of a Na+-coupled sialic acid symporter reveals a new Na+ site Nature Communications 9; 1753 doi: 10.1038/s41467-018-04045-7

6. Ferrandino G, Nicola JP, Sánchez YE, et al. 2016 Na+ coordination at the Na2 site of the Na+/I- symporter. Proc Natl Acad Sci U S A. 113:E5379-88.

7. Zhekova HR, Sakuma T, Johnson R, et al. 2020 Mapping of Ion and Substrate Binding Sites in Human Sodium Iodide Symporter (hNIS) J Chem Inf Model; 60: 1652-1665.

8. De la Vieja A, Reed MD, Ginter CS, et al. Amino acid residues in transmembrane segment IX of the Na+/I- symporter play a role in its Na+ dependence and are critical for transport activity. J Biol Chem. 2007 282:25290-8.

9. Levy O, De la Vieja A, Ginter CS, et al. 1998 N-linked glycosylation of the thyroid Na+/I- symporter (NIS). Implications for its secondary structure model. J Biol Chem.;273:22657-63.

10. Ravera S, Reyna-Neyra A, Ferrandino G, et al. 2017 The Sodium/Iodide Symporter (NIS): Molecular Physiology and Preclinical and Clinical Applications. Annu Rev Physiol 79:261-289.

11. Martín M, Bernal Barquero CE, Geysels RC, et al. 2019 Novel Sodium/Iodide Symporter Compound Heterozygous Pathogenic Variants Causing Dyshormonogenic Congenital Hypothyroidism. Thyroid 29:1023-1026.

12. Stoupa A, Al Hage Chehade G, et al. 2020 First case of fetal goitrous hypothyroidism due to SLC5A5/NIS mutations. Eur J Endocrinol. 183:K1-K5.

13. Reyna-Neyra A, Jung L, Chakrabarti M, et al. 2021 The Iodide Transport Defect-Causing Y348D Mutation in the Na(+)/I(-) Symporter Renders the Protein Intrinsically Inactive and Impairs Its Targeting to the Plasma Membrane. Thyroid. 2021 Jun 4. doi: 10.1089/thy.2020.0931.

14. Zhang CX, Zhang JX, Yang L, et al. 2021 Novel Compound Heterozygous Pathogenic Mutations of SLC5A5 in a Chinese Patient With Congenital Hypothyroidism. Front Endocrinol (Lausanne). 2021;12:620117

15. Martín M, Modenutti CP, Gil Rosas ML, et al. 2021 A Novel SLC5A5 Variant Reveals the Crucial Role of Kinesin Light Chain 2 in Thyroid Hormonogenesis. J Clin Endocrinol Metab. 106:1867-1881.

16. Watanabe Y, Ebrhim RS, Abdullah MA, et al. 2018 A Novel Missense Mutation in the SLC5A5 Gene in a Sudanese Family with Congenital Hypothyroidism. Thyroid. 28:1068-1070.
